# Assessment of the Effect of Leonurine Hydrochloride in a Mouse Model of PCOS by Gene Expression Profiling

**DOI:** 10.3390/genes15040507

**Published:** 2024-04-18

**Authors:** Mengmeng Wang, Li Yang, Guojie Sun, Yongbin Shao, Yuran Liu, Huiying Yang, Yan Wang, Mengyuan Zhang, Yunxia Shang, Xinli Gu

**Affiliations:** 1 College of Animal Science and Technology, Shihezi University, Shihezi 832000, China; mw1879976@163.com (M.W.); 15739330171@163.com (G.S.); 13381165329@163.com (Y.S.); 18636643042@163.com (Y.L.); 18337580359@163.com (H.Y.); 18509932185@163.com (Y.W.); 18899598896@163.com (M.Z.); 19109936506@163.com (Y.S.); 2 College of Veterinary Medicine, Xinjiang Agricultural University, Urumqi 830052, China; 15609931305@163.com

**Keywords:** leonurine hydrochloride, polycystic ovary syndrome, dehydroepiandrosterone, transcriptome

## Abstract

Polycystic ovary syndrome (PCOS) is an endocrine disease commonly associated with metabolic disorders in females. Leonurine hydrochloride (Leo) plays an important role in regulating immunity, tumours, uterine smooth muscle, and ovarian function. However, the effect of Leo on PCOS has not been reported. Here, we used dehydroepiandrosterone to establish a mouse model of PCOS, and some mice were then treated with Leo by gavage. We found that Leo could improve the irregular oestros cycle of PCOS mice, reverse the significantly greater serum testosterone (T) and luteinising hormone (LH) levels, significantly reduce the follicle-stimulating hormone (FSH) level, and significantly increase the LH/FSH ratio of PCOS mice. Leo could also change the phenomenon of ovaries in PCOS mice presented with cystic follicular multiplication and a lacking corpus luteum. Transcriptome analysis identified 177 differentially expressed genes related to follicular development between the model and Leo groups. Notably, the cAMP signalling pathway, neuroactive ligand-receptor interactions, the calcium signalling pathway, the ovarian steroidogenesis pathway, and the *Lhcgr*, *Star*, *Cyp11a*, *Hsd17b7*, *Camk2b*, *Calml4,* and *Phkg1* genes may be most related to improvements in hormone levels and the numbers of ovarian cystic follicles and corpora lutea in PCOS mice treated by Leo, which provides a reference for further study of the mechanism of Leo.

## 1. Introduction

Polycystic ovary syndrome (PCOS) is one of the most common endocrine diseases in women. The aetiology of PCOS is complex, and this disease mainly manifests as reproductive disorders, endocrine abnormalities, metabolic disorders, and other effects that have a great impact on female reproductive health [1]. Almost 10% of women of childbearing age suffer from anovulatory infertility and hyperandrogenaemia caused by PCOS [2]. Abnormal follicular development and an inability to produce mature egg cells are the main reasons for the reduced fertility and increased risk of miscarriage exhibited by women with this disease [3,4,5]. Additionally, most patients show manifestations of various metabolic abnormalities, such as dyslipidaemia and insulin resistance [6]. Currently, various theories to explain the pathogenesis of PCOS have been proposed, and these include genetic factors, neuroendocrine disorders, abnormal insulin secretion [7], disordered androgen metabolism disorders, defects in cortisol and lipid metabolism [8], chronic subclinical inflammatory processes, environmental factors, and psychological factors [9]. Although there is no cure for PCOS, new drugs to improve the symptoms of PCOS should be pursued.

Leonurine hydrochloride (Leo, 3,5-dimethoxy-4-hydroxybenzoic acid (4-guanidine)-1-butyl ester) is isolated as an alkaloid and is one of the main active ingredients of Leonurus [10]. Due to its anti-inflammatory [11,12], antioxidant, anti-apoptotic [13,14], and immune functions, an increasing number of researchers have focused on Leo. The good protective effect of Leo on cerebrovascular and cardiovascular diseases has been proven by pharmacological studies [15,16], and recent studies have shown that Leo also has antitumour, antidepressant, and oestrogen-like effects [17,18]. In addition, in terms of female animal reproduction, Leo could improve the development of pig embryos cultured in vitro by regulating mitochondrial function and oxidative stress levels [19] and could promote the maturation of bovine oocytes and embryonic development [20]. Leo has been shown to regulate the NLRP3/GSDMD signalling pathway, restore serum hormone levels in a cyclophosphamide-induced mouse model, and change the number of follicles at different stages of development; these effects can protect against premature ovarian failure [21]. All the abovementioned studies have shown the potential protective effect of Leo on female reproduction, indicating the therapeutic potential of Leo for reproductive diseases. In addition, Leo has low toxicity and good pharmacokinetic characteristics, and its clinical application prospects are very broad [22]. However, the effects of Leo on PCOS have rarely been reported. In this study, a mouse model of PCOS was established with dehydroepiandrosterone (DHEA), and some mice were then treated with Leo. The effects of Leo on the serum testosterone (T), luteinising hormone (LH), follicle-stimulating hormone (FSH), and LH/FSH levels and the numbers of cystic follicles and corpora lutea in the ovaries of PCOS mice were detected. High-throughput sequencing combined with bioinformatics analysis was then used to obtain differentially expressed genes (DEGs) between the ovaries of mice in the model group and Leo group, and the functions of these DEGs were preliminarily analysed. This study provides a theoretical basis for further study of the mechanism of Leo in PCOS.

## 2. Materials and Methods

### 2.1. Animals

Eighteen female C57BL/6 mice (aged 21 days) were provided by the Zhengzhou Huiji Huaxing Laboratory Animal Center (Zhengzhou, China). All the mice were raised in the Animal Center of Shihezi University with a 12 h light-dark cycle, a constant temperature of 25 °C, and a relative humidity of 50%. All mice were given adequate food and clean water. Six mice were randomly selected and placed in the same cage. To avoid the potential influence of the cage location on the experimental results due to differences in light intensity, the cages housing all the mice were placed side by side on the same horizontal plane in close proximity. All animal experiments were conducted in accordance with the standards of the Bioethics Committee of Shihezi University (approval number: A2022-75). These experiments were conducted using an unregistered protocol, and all the participants were aware of the group allocations at different stages of the study.

### 2.2. PCOS Model Construction and Leo Treatment

Induction of PCOS in twelve mice was achieved by subcutaneous injection of DHEA (6 mg/100 g body weight, dissolved in 90 μL of sesame oil with 10 μL of 95% alcohol) for 20 consecutive days [23], and the success of the established PCOS model was judged by detecting the oestrus cycle [24]. On average, the successfully constructed PCOS mice were randomised into two groups: the mice in the Leo group were gavaged with Leo (20 mg/kg body weight, purity above 98%, purchased from Shanghai Yuanye Biotechnology Co., Ltd., Shanghai, China) for 20 consecutive days [21], and those in the PCOS group were gavaged with the same amount of normal saline. The mice in the control group were subcutaneously injected with 100 μL of sesame oil containing 10 μL of 95% ethanol for 20 consecutive days and then gavaged with 0.2 mL of normal saline for 20 days.

### 2.3. Detection of the Oestrus Cycle

Starting from the fifth day after DHEA injection (26 to 41 days of life) and the fifth day after intragastric administration (46 to 61 days of life), the vagina of each mouse was rinsed with normal saline, and vaginal cells were collected at 9 a.m. every day. After staining with haematoxylin and eosin (H&E) and microscopic examination, the stages of the oestrus cycle were determined according to previously described methods [24,25].

### 2.4. Determination of Serum Hormones

After 20 days of Leo treatment, all the mice were treated humanely according to the standards of the Bioethics Committee of Shihezi University. Blood was collected from all the mice, and serum was separated. The FSH, LH and T levels were assayed using ELISA kits, and the operation followed the manufacturer’s instructions (Solabia, Beijing, China).

### 2.5. Testing of Ovarian Tissue

Three mice were randomly selected from each group, and paraformaldehyde was used for 24 h to fix the bilateral ovarian tissues. Serial paraffin sections of the ovaries were then cut at 5 µm thickness, and to analyse the number of corpora lutea and cystic follicles, 1 of every 10 sections was stained with H&E [26]. The ovaries were frozen at −80 °C for the three remaining mice in both the model and Leo groups, and the left ovaries were selected for transcriptome sequencing.

### 2.6. Library Construction and Sequencing

Total RNA was extracted from the left ovaries of mice in the model and Leo groups using TRIzol (Invitrogen, Carlsbad, CA, USA) according to the manufacturer’s instructions. RNA concentration and purity were assessed using an RNA Nano 6000 Assay Kit (Agilent Technologies, Santa Clara, CA, USA). Agarose gel electrophoresis coupled to an Agilent 2100 bioanalyser was used to assess RNA integrity and total RNA content. The Ribo-zero™ rRNA Removal Kit (Epicentre, Madison, WI, USA) was used to remove ribosomal RNA. Next, sequencing libraries were generated using the NEBNext Ultra™ Directional RNA Library Prep Kit (Illumina, Bellingham, NE, USA). An AMPure XP system (Beckman Coulter, Beverly, CO, USA) was used to obtain cDNA fragments of 150–200 bp. The cDNA of selected length was treated with the USER enzyme (NEB, Ipswich, MA, USA), followed by PCR amplification using random primers and Phusion High-Fidelity DNA Polymerase. An Agilent 2100 bioanalyser was used for library identification. The library was then sequenced on the Illumina NovaSeq 6000 platform. Paired-end reads were generated for further analysis.

### 2.7. Sequence Processing and Analysis

To ensure the quality of the results of subsequent bioinformatics analysis, we used FastQC software (v0.11.8) to perform quality control on the raw data. To obtain high-quality reads, reads with *n* > 10% (*n* represents the undetermined base content) were removed, and reads that contained linker sequences or for which data for more than half of the bases were of low quality (Phred score < 5%) were also removed. These high-quality reads were defined as clean reads for subsequent analysis. In addition, the FastQC software was used to measure the Q30 value, which estimates the quality of the sequencing data for further quality control. The clean data were aligned with the mouse reference genome using HISAT2 (v2.0.5) [27]. The transcripts from the reads in each sample alignment were assembled into splicing transcripts using Cufflinks (v2.0.2) software [28]. Cuffmerge (v2.0.2) was used to deduplicate genes containing multiple transcripts and select the longest transcript as a unigene, thereby obtaining the unigene set of each sample, and this dataset was used for subsequent analysis [29].

### 2.8. Identification of Differentially Expressed Genes

After assembling and screening high-quality transcript data as described above, we used Cuffdiff software to quantify these transcripts for differentially expressed genes (DEGs) analysis [30]. The values of fragments per kilobase of the exon model per million mapped fragments (FPKM) were used for the normalisation of gene expression in different samples [31]. After obtaining the FPKM values of each sample, we used the negative binomial distribution model in the DESeq2 R package (1.16.1) to identify the DEGs. The Benjamini and Hochberg method was adopted to adjust the corrected *p* value, and genes with significant differential expression were screened based on the |log2foldchange| value. We used RNASeqPower to calculate the power of these samples in the test.

### 2.9. Functional Enrichment Analysis of the DEGs

A Gene Ontology (GO) analysis was conducted using hypergeometric test *p* values to analyse the enrichment of the DEGs. The differentially expressed gene sets were mapped to each term in the GO database to analyse the number of genes that were mapped to biological process (BP), cellular component (CC), and molecular function (MF) terms. The Bonferroni method was used to correct the *p* values. A GO term was deemed significantly enriched if its corrected *p* values were lower than 0.05 [32]. The ClusterProfiler (3.4.4) R package was used to perform a Kyoto Encyclopedia of Genes and Genomes (KEGG) (http://www.genome.jp/kegg/, accessed on 13 October 2020) pathway enrichment analysis of the DEGs. Significantly enriched pathways were screened according to the hypergeometric test [33]. The criterion for significant enrichment was set at *p* < 0.05.

### 2.10. Differential Gene Expression Analysis and RT-qPCR Validation

To ensure the accuracy of the transcriptome database, we randomly selected 8 DEGs to quantify their expression levels. Based on GO and KEGG enrichment analyses, DEGs related to follicle development were screened and selected for quantification of their expression levels. All primers were designed using Primer 5 software (v5.00) (Supplementary Table S1). According to the above-described method, RNA was extracted, and cDNA was synthesised from 1 μg of RNA via a reverse transcription kit (Takara, China). We monitored product formation by RT-qPCR with the fluorescent dye SYBR Green (Takara Biotech, Dalian). β-Actin was used as an endogenous control. The RT-PCR system included 7.4 μL of ddH_2_O (RNase-free), 1 μL of template cDNA, 10 μL of SYBR Green PCR Master Mix (Takara, China), 0.8 μL of the upstream primer, and 0.8 μL of the downstream primer (gene primer sequences are shown in Table S1). All reactions were repeated three times and performed under the following conditions: 35 cycles of 94 °C for 10 s, 57 °C for 10 s, and 72 °C for 20 s. After the reaction, gene expression was calculated as E = 2^−ΔCt^ based on the critical threshold cycle (CT) value of each reaction [34].

### 2.11. Statistical Analysis

Descriptive statistics and one-way analysis of variance were performed to analyse the hormone levels and RT-qPCR results using IBM SPSS Statistics 26 software. The results are expressed as the means ± standard deviations, and a *p* value ≤ 0.05 was considered to indicate a statistically significant difference. No data from animals that met the inclusion/exclusion criteria appeared outside the abovementioned test range.

## 3. Results

### 3.1. Effects of Leo on the Oestrous Cycle in Mice

**Figure 1:**
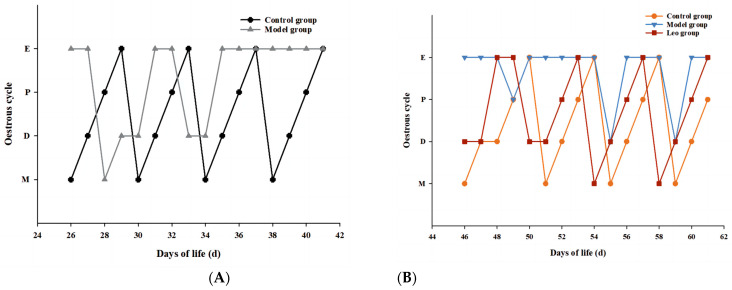
Results of the oestrous cycle. (**A**) Changes in the oestrous cycle of mice after DHEA injection between the control and model groups. (**B**) Changes in the oestrous cycle among the control, model, and Leo mice after Leo treatment.

After subcutaneous injection of DHEA, the mice showed a disturbance of the oestrus cycle with prolonged oestrus, whereas the control mice maintained a normal oestrus cycle (Figure 1A). This result indicated the successful construction of a mouse model of PCOS. After Leo treatment, the oestrous cycle of the mice returned to normal (Figure 1B).

### 3.2. Effects of Leo on the Serum Hormone Levels in the PCOS Mouse Model

**Figure 2:**
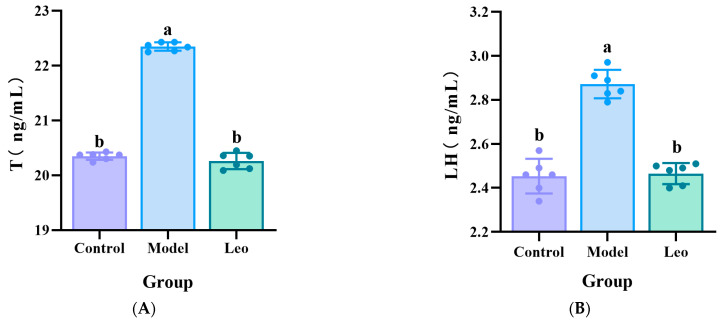

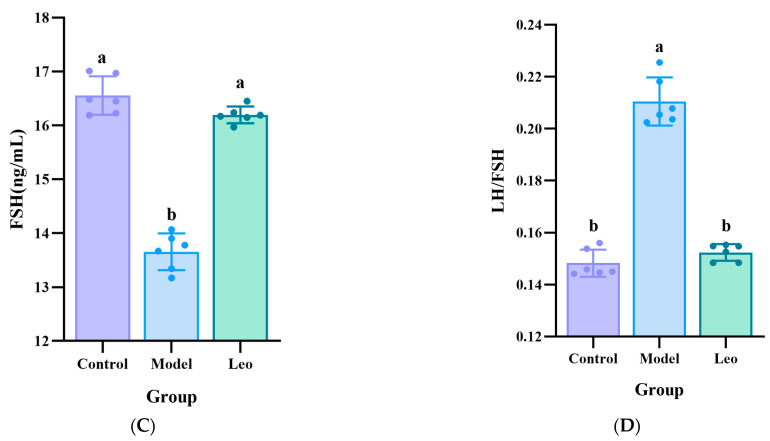
Results of the analysis of the hormone levels. (**A**) Serum T levels in the three groups of mice. (**B**) Serum LH levels in the three groups of mice. (**C**) Serum FSH levels in the three groups of mice. (**D**) Serum LH/FSH values of the three groups of mice. The letters a and b denote different shoulder markers: different shoulder markers indicate significant differences, and the same shoulder markers indicate nonsignificant differences.

To further confirm the effect of Leo on PCOS mice, the serum levels of T, LH, and FSH were measured in the mice of the three groups using ELISA. The results showed that the model group exhibited significantly greater T and LH levels, a significantly lower FSH level, and a greater LH/FSH ratio compared to the control group. Treating with Leo could decrease the T and LH levels, increase the FSH levels, and decrease the LH/FSH values that did not significantly differ from those of the control group (Figure 2A–D), which indicated that Leo may improve the serum hormone levels that exhibit abnormal changes in PCOS.

### 3.3. Effects of Leo on the Ovaries of PCOS Model Mice

**Figure 3:**
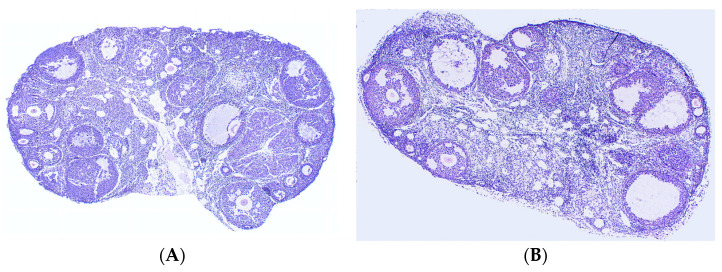

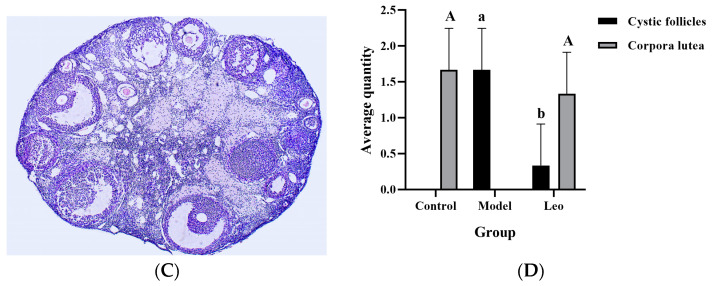
Results from analyses of sections of mouse ovaries. (**A**) H&E staining of ovarian sections from control mice. (**B**) H&E staining of ovarian sections from model mice. (**C**) H&E staining of ovarian sections from mice in the Leo group. (**D**) Statistics of the numbers of cystic follicles and corpora lutea in the ovaries from three groups of mice. When comparing cystic follicles, the letters a and b denote different shoulder markers: different shoulder markers indicate significant differences, and the same shoulder markers indicate nonsignificant differences. When comparing corpora lutea, the letters A and B denote different shoulder markers: different shoulder markers indicate significant differences, and the same shoulder markers indicate nonsignificant differences.

Ovarian sections from mice in the three groups were stained with H&E, and the number of cystic follicles and corpora lutea was counted to determine the effect of Leo on the ovary of PCOS model mice. In comparison to the control group (Figure 3A), the mice in the model group had an increase in the number of cystic follicles and a decrease in the number of corpora lutea (Figure 3B). Although there were still differences in ovarian morphology between the Leo group (Figure 3C) and the control group, Leo could reverse the ovarian changes in the model mice to a certain extent. Figure 3D shows the results of statistical analyses of the numbers of corpora lutea and cystic follicles in the mouse ovaries of the three groups.

### 3.4. Basic RNA-Seq Data Analysis

**Figure 4:**
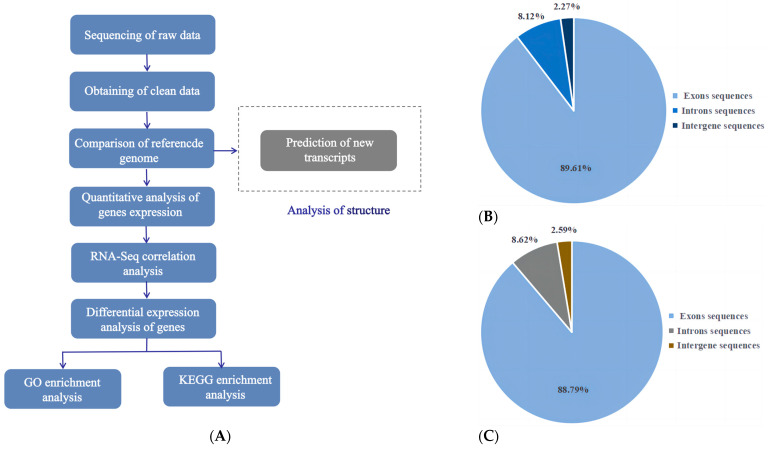
Basic bioinformatics analysis pipeline and results. (**A**) Bioinformatics analysis pipeline of the mouse ovarian transcriptome sequencing results. (**B**) Pie chart of the statistics of the alignment of clean reads from the model group to the reference genome. (**C**) Pie chart of the statistics of the alignment of clean reads from the Leo group to the reference genome.

To understand how Leo alters serum hormone levels in mice with PCOS, reduces the number of cystic follicles in the ovaries, and increases the number of corpora lutea, we analysed the gene expression profiles of the ovaries of mice in the model and Leo groups using transcriptome technology. The Illumina NovaSeq 6000 platform was used for paired-end RNA sequencing to detect patterns of gene expression in the ovaries of the two different groups of mice. The sequencing data were analysed according to the pipeline shown in Figure 4A. Overall, we obtained 260,929,152 raw reads in this transcriptome analysis. To improve the accuracy of subsequent bioinformatics analysis, FastQC was used to perform quality control on these raw reads. After processing using the quality control process described in the Methods section, we obtained a total of 253,136,448 clean reads for subsequent analysis. The data efficiency was greater than 97.00%. The quality of these data was further evaluated; high-quality data with Phred values greater than 30 (Q30) accounted for more than 94.29% of the total data, and the GC content was 49.97%. The study’s results showed that the sequencing quality was good and that the data met the requirements for further analyses. Subsequently, the mouse reference genome was compared with the clean data. This analysis showed that 89.61%, 8.12%, and 2.27% of the RNA-seq data of the model group mapped to exon, intron, and intergene sequences, respectively (Figure 4B), and 88.79%, 8.62%, and 2.59% of the RNA-seq data of the Leo group mapped to these three regions, respectively (Figure 4C). In total, 47 novel genes were found after in-depth analysis (Supplementary Table S2). The RNASeqPower values of the samples in both groups were 100%.

### 3.5. Identification of DEGs

**Figure 5:**
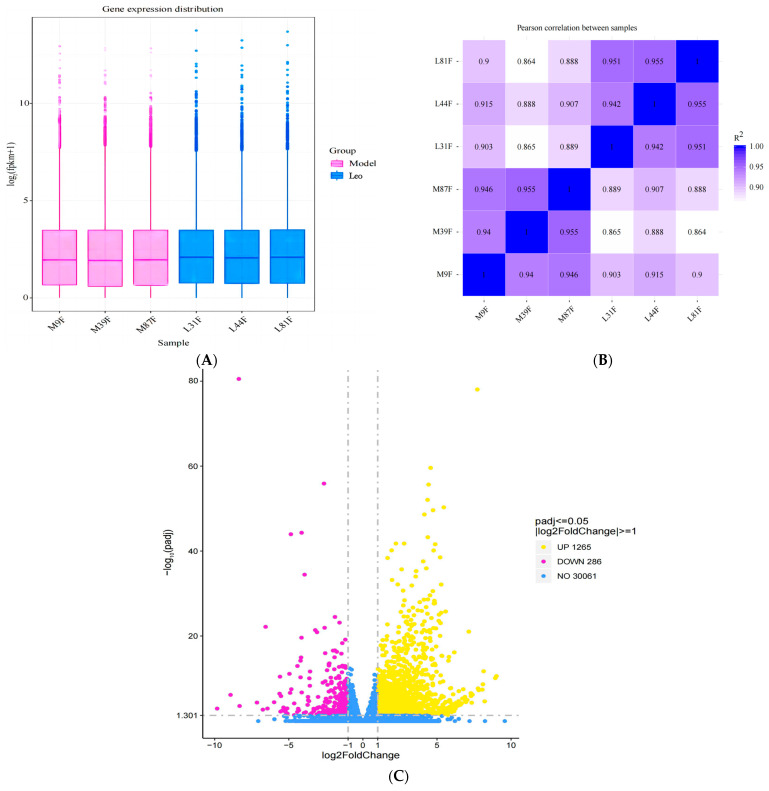
Results of the DEG analyses. (**A**) The box plots display the distributions of gene expression levels across various samples. The figure displays the sample name on the abscissa and the log_2_ (FPKM+1) value on the ordinate. Each box plot represents five statistics, namely the maximum value, upper quartile, median, lower quartile, and minimum value, from top to bottom of the box. (**B**) Heatmap of the correlations between samples. The squares of the correlation coefficients for each sample are shown as horizontal and vertical coordinates. (**C**) Volcano plot of DEGs between the Leo group and the model group.

The total number of reads mapped (including multiple mapped and uniquely mapped reads) to the reference sequence was calculated using the clean reads, along with the corresponding percentages among them. Thus, the gene expression levels in the ovaries of mice in the model group and Leo group were calculated. To rule out bias based on the gene length and sequencing depth, we used the FPKM method to normalise the mRNA expression levels. The DEGs of the different groups were screened according to the normalised FPKM value. Gene expression patterns across the different samples were relatively uniform across the two groups of mouse ovaries after the different treatments (Figure 5A). Additionally, we performed a correlation analysis to judge the repeatability between samples. The results showed that the smallest R2 value between these samples was 0.86, which is greater than 0.8 (Figure 5B) [35] and thus indicates that the samples exhibit good biological repeatability.

Subsequently, the gene expression levels in the two groups of differently treated samples were analysed, and we found that 1551 genes exhibited differential expression in the Leo group compared with the model group (Padj < 0.05); these genes included 1265 upregulated genes and 286 downregulated genes (Figure 5C, Supplementary Table S3). Further analysis of the DEGs indicated that the expression of some genes that regulate follicle development showed opposite patterns in the two groups. Therefore, these DEGs may be target genes by which Leo improved the T, LH, and FSH levels and follicle development in the PCOS mouse model.

### 3.6. Functional Enrichment Analysis of the DEGs

**Figure 6:**
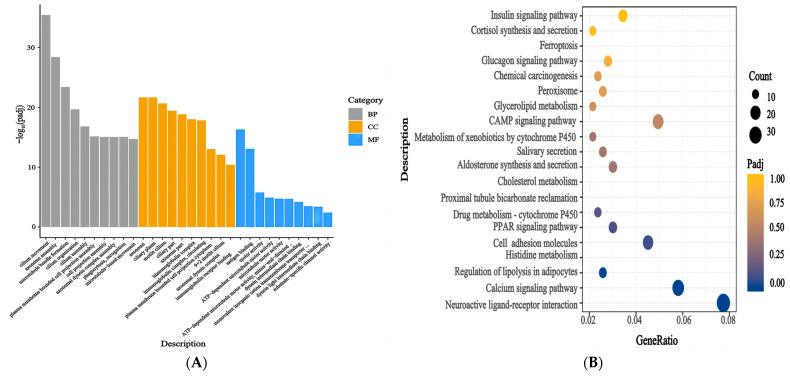
Functional enrichment analysis of DEGs. (**A**) GO annotation results for the top 30 DEGs between the two groups. The graph displays the GO terms on the *x*-axis and their significance level of enrichment (−log10(Padj)) on the *y*-axis. Functional categories are distinguished by different colours. (**B**) The top 20 pathways from the KEGG enrichment analysis of DEGs between the two groups.

The enrichment of the DEGs in the ovaries of mice in the Leo and model groups was analysed using the GO and KEGG databases. The GO annotation results indicated that 776 GO terms were significantly enriched in the DEGs (*p* < 0.05). These terms included 134 MF terms, 69 CC terms, and 573 BP terms (Supplementary Table S4). The top 30 significantly enriched terms included axoneme assembly, the axoneme, membrane invagination, and other terms related to follicle development (Figure 6A). This result suggests that a large degree of membrane invagination and many immune-related genes may be important for follicular development. We then performed enrichment analysis of the DEGs using the KEGG database. The biological functions of genes were analysed according to the functions of their gene products. The results indicated that 43 pathways were significantly enriched (*p* < 0.05) (Supplementary Table S5), and these included cell adhesion molecules, the PPAR signalling pathway, cholesterol metabolism, the calcium signalling pathway, aldosterone synthesis and secretion, melanogenesis, neuroactive ligand-receptor interactions, histidine metabolism, lipolysis in adipocytes, the cAMP signalling pathway, and other signalling pathways (Figure 6B). These results suggested that the ovaries of the PCOS mice exhibited a strong biological response to Leo treatment.

### 3.7. Differential Gene Expression Analysis and RT-qPCR Validation

**Figure 7:**
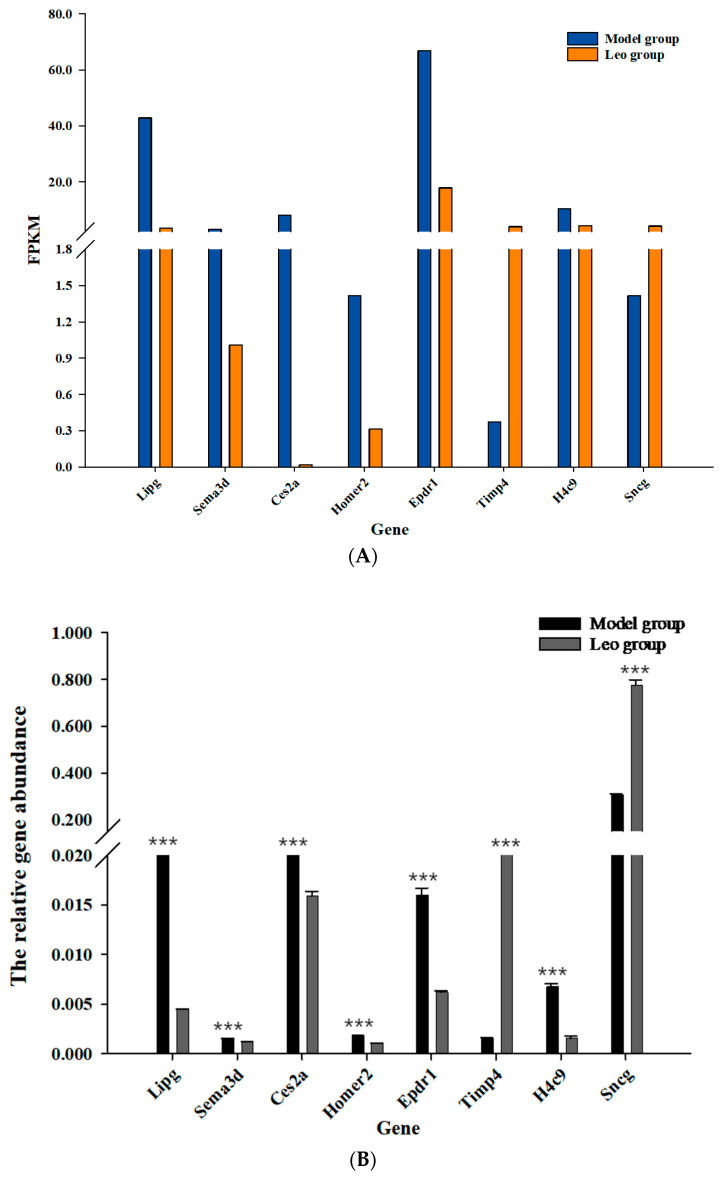
RT-qPCR validation of DEGs in the ovarian RNA-seq database in the model and Leo groups. (**A**) The transcript abundance of DEGs was calculated using the FPKM method. (**B**) RT-qPCR validation of the expression levels of DEGs. *** *p* < 0.001.

The accuracy of the RNA-seq results is crucial to their analysis; thus, we verified the DEGs in the ovaries of the mice in the two groups using molecular biology. Eight DEGs were randomly selected for verification of their expression levels. First, we examined the RT-qPCR amplification products by agarose gel electrophoresis and observed a single band of the expected size. Additionally, DNA sequencing of these RT-qPCR products indicated that the amplification products were accurate. These results indicated that the designed primers were accurate and specific and met the requirements for RT-qPCR analysis. RT-qPCR was used to analyse the expression levels of eight DEGs in the ovaries of mice in the two groups. *Lipg*, *Sema3d*, *Ces2a*, *Homer2*, *H4c9,* and *Epdr1* were significantly downregulated in the Leo group (*p* < 0.05), and *Timp4* and *Sncg* were significantly upregulated in the Leo group (*p* < 0.05). However, these genes showed opposite expression patterns in the model group. The results showed the same changes as those detected by RNA-Seq (Figure 7, Supplementary Tables S6 and S7). Thus, the gene expression levels in the mouse ovaries were accurately reflected by the RNA-seq data.

### 3.8. Analysis of DEGs Most Relevant to Ovary and Validation by RT-qPCR

**Figure 8:**
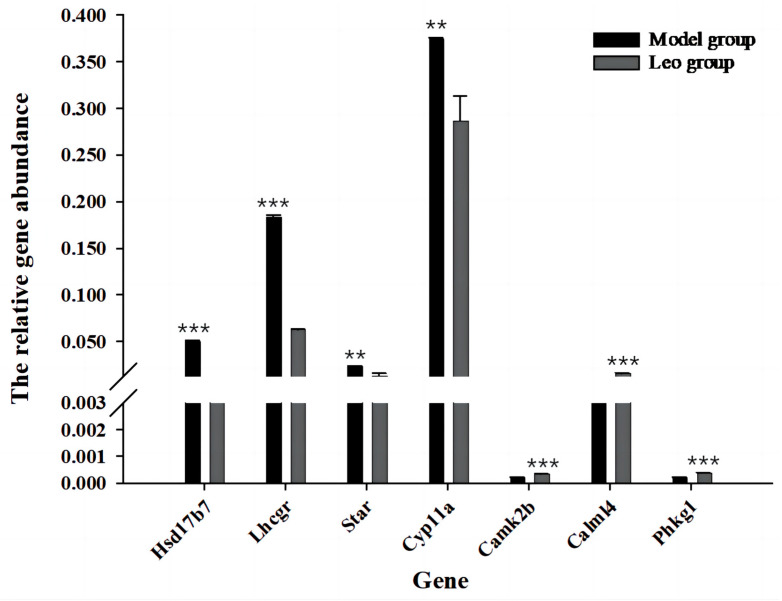
RT-qPCR validation of the DEGs between the model and Leo groups most relevant to the ovary. RT-qPCR was used to verify the expression levels of the DEGs most relevant to the ovary. ** *p* < 0.01, *** *p* < 0.001.

Further analysis of the abovementioned significantly enriched signalling pathways revealed that 10 of the identified signalling pathways may be related to follicle development (Supplementary Table S8); among these pathways, the neuroactive ligand-receptor interaction pathway [mmu04080] (36 DEGs, *p* = 7.73 × 10^−5^) was enriched in the highest number of DEGs and exhibited the most significant difference. The calcium signalling pathway [mmu04020] (27 DEGs, *p* = 8.59 × 10^−5^) and cAMP signalling pathway [mmu04024] (23 DEGs, *p* = 0.005067676) were enriched in the next highest numbers of DEGs. The other pathways were cell adhesion molecules [mmu04514] (21 DEGs, *p* = 0.000580393), the PPAR signalling pathway [mmu03320] (14 DEGs, *p* = 0.000807598), aldosterone synthesis and secretion [mmu04925] (14 DEGs, *p* = 0.003713544), ovarian steroidogenesis [mmu04913] (8 DEGs, *p* = 0.032427366), GnRH secretion [mmu04929] (8 DEGs, *p* = 0.045764218), and other signalling pathways. These pathways were enriched in a total of 177 DEGs, which included 131 upregulated genes and 46 downregulated genes. Among these, *Hsd17b7*, *Star*, *Cyp11a1*, *Lhcgr*, *Camk2b*, *Calml4*, and *phkg1* exhibited the strongest relationship to ovarian development. RT-qPCR revealed that the Leo group exhibited significantly downregulated expression of *Hsd17b7*, *Star*, *Cyp11a1,* and *Lhcgr* (*p* <0.05) and significantly upregulated expression of *Camk2b*, *Calml4,* and *phkg1* compared with the model group (*p* < 0.05) (Figure 8).

## 4. Discussion

PCOS is a common endocrine disorder that greatly affects women of reproductive age. Although the diagnostic criteria for PCOS have not been unified, a study conducted by the Androgen Excess Society (AES) showed that excess androgen or hyperandrogenism is an important index for the diagnosis of the disease, and this characteristic is included as an essential element of all “consensus” diagnostic protocols [36,37]. In addition, the ovaries of patients with PCOS often exhibit an increased number of cystic follicles [38,39]. In this study, mice in the model group showed higher T and LH levels, increased numbers of cystic follicles in the ovaries, decreased FSH and LH/FSH values, and a decreased number of corpora lutea compared with those in the control group [25,40]. These results showed the successful establishment of the PCOS mouse model. The PCOS mouse model was successfully established [41,42]. Interestingly, Leo treatment reversed the changes in the T, LH, FSH, and LH/FSH levels in PCOS mice. Although some differences in ovarian morphology were noted between the Leo group and the control group, the ovaries of mice in the Leo group showed significantly lower numbers of cystic follicles and a higher number of corpora lutea compared with the ovaries of mice in the model group. These results indicated that Leo was effective in alleviating the abnormalities in the T, LH, and FSH levels and follicle development in PCOS mice, suggesting that this treatment improved these parameters in mice with DHEA-induced PCOS. To further analyse how Leo affected hormone levels and follicular development in PCOS mice, RNA-seq was used to detect changes in the gene expression profiles of the ovaries of mice in the two groups, and RT-qPCR was used to analyse the accuracy of the sequencing results. The samples showed good biological repeatability, and the sequencing data were of good quality. The identified DEGs truly showed changes in their expression levels in mouse ovaries, and subsequent bioinformatics analysis could refer to sequencing data.

A total of 177 DEGs related to ovarian development were identified in the Leo group compared with the model group; of these genes, 131 DEGs were upregulated and 46 DEGs were downregulated, indicating that the ovaries of the mice with DHEA-induced ovulatory dysfunction exhibited a strong biological response to Leo treatment. These DEGs were significantly enriched in GO terms that included axoneme assembly, the axoneme, membrane invagination, and other terms related to follicular development. These DEGs were significantly enriched in 10 signalling pathways related to follicular development, suggesting that the ability of Leo to improve DHEA-induced PCOS in mice involves the regulation of multiple biological pathways. Among these pathways, neuroactive ligand-receptor interactions were enriched in the highest number of DEGs and showed the most significant differences, followed by the calcium signalling pathway and the cAMP signalling pathway. Studies have shown that interactions between neuroactive ligands and receptors are involved in granulosa cell proliferation [43], and these interactions, along with signalling pathways such as the PPAR and cAMP signalling pathways, are important for ovarian development [44] and are related to the occurrence and development of PCOS [45,46]. The calcium signalling pathway is crucial in regulating various cellular processes such as cell growth, differentiation, proliferation, apoptosis, and other cellular processes [47]. Previous studies have shown that calcium acts as a second messenger in most species and all mammals, that calcium signalling can restore oocyte meiosis and activate the egg, which is essential for the regulation of embryonic development [48,49], and that the activation of calcium signalling pathways can inhibit follicular atresia [50]. cAMP signals can regulate the excitability of gonadotropin-releasing hormone (GnRH) neurons [51,52]. GnRH regulates the levels of female reproductive hormones such as luteinising hormone, which affects ovulation. These results indicated that these three pathways may be most strongly related to the abnormalities in the T, LH, and FSH levels and numbers of cystic follicles and corpora lutea in PCOS mice regulated by Leo.

In addition, the luteinising hormone chorionic gonadotropin receptor (*Lhcgr*) gene was enriched in four signalling pathways, including neuroactive ligand-receptor interaction, the calcium signalling pathway, the cAMP signalling pathway, and ovarian steroidogenesis, suggesting that *Lhcgr* is an important target gene of Leo in the treatment of PCOS mice. We also found that the Leo group exhibited significantly reduced expression of *Lhcgr*, steroidogenic acute regulatory protein (*Star*), recombinant cytochrome P450 11A1 (*Cyp11a*), and 17-β hydroxysteroid dehydrogenase type 7 (*Hsd17b7*) and significantly greater expression of calcium/calmodulin-dependent protein kinase II β (*Camk2b*), calmodulin-like protein 4 (*Calml4*), and phosphorylase kinase and γ 1 (*Phkg1*) compared with the model group. *Lhcgr*, *Star*, *Cyp11a,* and *Hsd17b7* are key genes that regulate the production of ovarian steroid hormones and have been identified as potential target genes in the occurrence and development of PCOS [53,54,55]. The expression of *Star* and *Cyp11a* is reportedly increased in ovarian tissues in PCOS [56,57], and the expression of *Lhcgr* is enhanced or overactivated [58,59,60], leading to increased androgen synthesis in the cells of the follicular membrane and resulting in disturbed hormone levels and the arrest of follicular maturation. Follicular development depends on oocyte maturation, granulosa cell proliferation, and granulosa cell differentiation. Oocyte maturation is also dependent on the supply of steroids and nutrients by granulosa cells [61]. Our findings suggest that Leo may activate the calcium signalling pathway by reducing the overexpression of *Lhcgr* and increasing the expression of calcium-related regulatory proteins such as *Camk2b*, *Calml4*, and *Phkg1* in the ovaries of PCOS mice. By regulating the expression of genes such as *Star*, *Cyp11a*, and *Hsd17b7*, Leo affects ovarian steroidogenesis [62], alters the levels of T, LH, and FSH, and regulates the cAMP signalling pathway by changing the expression of genes such as *SOx9*, thereby affecting changes in downstream related signalling pathways. Moreover, Leo ameliorated the abnormalities in the levels of T, LH, and FSH and the numbers of ovarian cystic follicles and corpora lutea in PCOS mice.

However, this study has several limitations, and in the future, we will continue to investigate the mechanism underlying the effect of Leo on PCOS mice.

## 5. Conclusions

In this study, we successfully established a PCOS mouse model and treated some of the mice with Leo. The results showed that Leo could not only ameliorate the irregular estros cycle of DHEA-induced PCOS mice but also improve T, LH, and FSH dysregulation, as well as the changes in ovarian cystic follicles and corpora lutea in PCOS mice. The signalling pathways and DEGs related to follicular development successfully screened by RNA-seq may be most relevant to Leo treatment of PCOS mice, which provides a good basis for further mechanism studies.

## Data Availability

All of the original sequences obtained in the current study have been deposited in the Sequence Read Archive (SRA) under BioProject number PRJNA884922. All of the data can also be obtained from the corresponding author upon reasonable request or from the included Supplementary Materials.

## Supplementary Materials

The following supporting information can be downloaded at: https://www.mdpi.com/article/10.3390/genes15040507/s1. Figure S1: Evaluation of RNA integrity by agarose gel electrophoresis, Figure S2: Validation of primer specificity by agarose gel electrophoresis, Table S1: Detailed information on primers used for all RT-qPCR in this study; Table S2: The information on novel transcripts predicted in this study; Table S3: Differentially expressed genes between the Leo group and the model group; Table S4: GO annotation of differentially expressed genes between the Leo group and the model group; Table S5: KEGG enrichment analysis of differentially expressed genes between the Leo group and the model group; Table S6: FPKM values of some differentially expressed genes; Table S7: The expression of genes analyzed by RT-qPCR.; Table S8: Ten signalling pathways associated with ovarian development.
