# Supplementary figures and images for: Assessment of the Effect of Leonurine Hydrochloride in a Mouse Model of PCOS by Gene Expression Profiling
Source: Genes (Basel). 2024 Apr 18;15(4):507. doi: 10.3390/genes15040507 (PMC11050333; doi:10.3390/genes15040507)

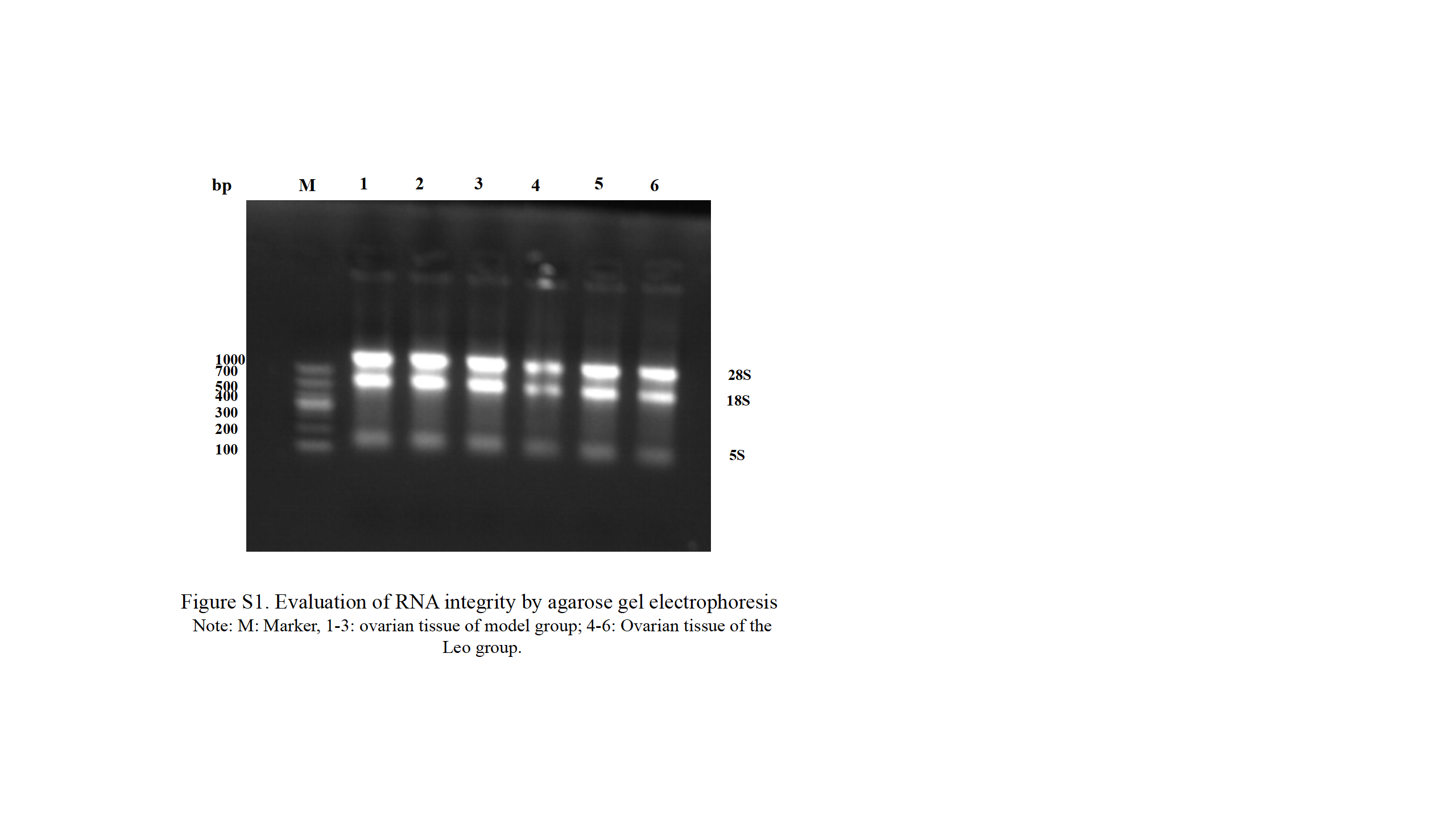

Supplement: Supplementary file 1 [file genes-15-00507-s001.zip › Figure S1 Evaluation of RNA integrity by agarose gel electrophoresis.png]

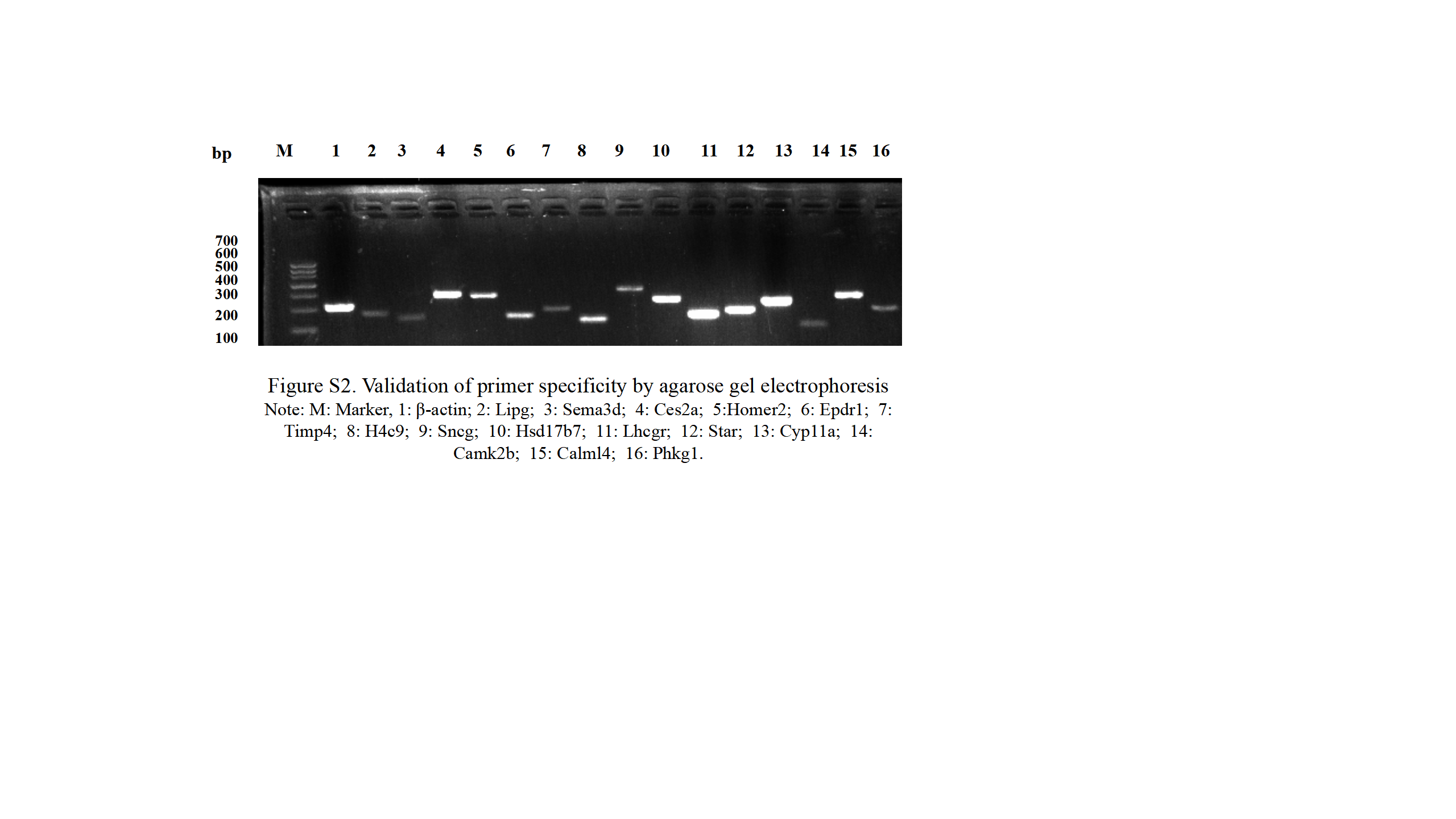

Supplement: Supplementary file 1 [file genes-15-00507-s001.zip › Figure S2 Validation of primer specificity by agarose gel electrophoresis.png]
